# Effects of Preoperative Carbohydrate Intake on Inflammatory Markers and Clinical Outcomes in Elderly Patients Undergoing Radical Prostatectomy: A Single-Centre, Double-Blind Randomised Controlled Trial

**DOI:** 10.3389/fsurg.2021.744091

**Published:** 2021-11-18

**Authors:** Zhen Hu, Ji Liu, Fen Wang

**Affiliations:** Shanghai Tenth People's Hospital, Tongji University, Shanghai, China

**Keywords:** carbohydrate, inflammatory markers, enhanced recovery after surgery (ERAS), clinical outcomes, radical prostatectomy

## Abstract

**Background:** This study aimed to analyse the effects of carbohydrate (CHO) intake on inflammatory markers, comfort, and clinical outcomes in elderly patients undergoing open radical prostatectomy.

**Methods:** Patients aged ≥65 years who underwent open radical prostatectomy were randomly divided into CHO, drinking water, and fasting groups. A total of 90 patients were enrolled in this study (CHO group, *n* = 28; placebo group, *n* = 30 and fasting group, *n* = 32). Patients in the CHO group were given 800 and 400 ml of carbohydrates 8 and 2–3 h before surgery, respectively. Patients in the placebo group were given 800 and 400 ml of water 8 and 2–3 h before surgery, respectively. Patients in the fasting group did not consume any liquids. The main result is levels of inflammation markers. Secondary results included cellular immunity, comfort, body weight, grip index, and clinical results.

**Results:** Compared with the fasting group, the CHO group exhibited a decrease in interleukin 6 (IL-6) levels on days 1 and 7 (75.47 and 7.06 pg/mL, respectively), IL-8 levels on day 1 (274.61 pg/mL) and tumour necrosis factor (TNF) levels on days 1, 3, and 7 (11.16, 9.55, and 9.67 pg/mL, respectively). The placebo group exhibited a decrease in IL-8 (390.26 pg/mL) and TNF levels (13.99 pg/mL) on day 1. Compared with the placebo group, the CHO group exhibited a decrease in IL-6 levels on day 1 and TNF levels on day 3. In the CHO and placebo groups, the thirst and hunger scores decreased on the morning of surgery.

**Conclusion:** Preoperative CHO and drinking water are associated with decreased levels of IL-6, IL-8, and TNF. CHO and water can also reduce thirst and hunger scores. Therefore, we recommend that patients without contraindications should be given 200–400 ml of fluid 2–3 h before surgery, preferably CHO.

**Clinical Trial Registration:** http://www.chictr.org.cn/edit.aspx?pid=21783&htm=4; ChiCTR-INR-17012867.

## Introduction

Patients who undergo radical prostatectomy are generally at an advanced age with multiple comorbidities. Surgical trauma generally leads to a longer recovery time; therefore, accelerated rehabilitation is required. Owing to the popularity of enhanced recovery after surgery (ERAS) (1, 2), administering preoperative oral carbohydrate (CHO) has become a common clinical practise (3, 4). Preoperative administration of CHO can reduce insulin resistance, protein loss, hunger, and anxiety in patients without affecting gastric emptying (5). CHO can promote early recovery of the intestinal function and shorten the hospitalisation period (6). Currently, the most common studies include assessment of the effects of CHO on insulin resistance (7) and comfort (8) and the effects of minimally invasive surgery (9) and unconventional fasting (10) on postoperative immune function. A few studies have been concerned with the improvement of postoperative immune function by CHO.

Major open abdominal or pelvic surgery has a higher incidence of postoperative adverse events such as cardiopulmonary insufficiency, pain, thromboembolic complications, and infection. The main reason for such complications is the stress response caused by surgical trauma, followed by a relatively high-level demand for a patient's immunity and energy reserve. The relatively high-level demand for a patient's organ function is considered to be mediated by endocrine and metabolic changes caused by trauma.

The levels of C-reactive protein (CRP) and cytokines are closely related to immune reaction, inflammatory response and the extent of the inflamed tissue. Interleukin (IL-6) levels are associated with the incidence of postoperative complications and are one of the predictors of the incidence of adverse events postoperatively.

We hypothesise that drinking fluids before surgery can improve the immune function of patients after surgery, and the level of certain important inflammatory factors has increased. The level of inflammatory factors has a certain warning effect on the outcome of patients (such as infection, etc.). Therefore, this study hypothesises that drinking liquids before surgery can improve the outcome of patients by regulating inflammatory factors.

## Methods

Patients who underwent open radical resection of prostate cancer in the Shanghai Tenth People's Hospital were selected for this study. The inclusion criteria were as follows: elective radical resection of prostate cancer; age ranging from 65 to 85 years; body mass index (BMI) ranging from 17.0 to 32.0 kg/m^2^; the American Society of Anesthesiologists (ASA) physical status I-III and normal heart, lung, liver, kidney, and blood coagulation function. Oral anticoagulants were discontinued 5–7 days before the operation. The exclusion criteria were as follows: age <65 years, inability to drink transparent liquid or allergy, gastrointestinal emptying disorder or obstruction, diabetes, liver cirrhosis, severe cardiac and renal insufficiency, corticosteroid administration at a dose more than 5 mg/day, and ASA physical status IV. The trial was approved by the Ethics Committee of the Shanghai Tenth People's Hospital, and all patients signed a written informed consent form before participating in the study.

All patients were randomly divided into the following three groups: CHO, water (placebo group) and routine water abstinence groups (fasting group). The patients were divided as follows: According to the required sample size of 120 patients, 120 two-digit random number series were generated using a random number table. The order of the remainder obtained by dividing the two-digit random number series by 3 was the order in which the patients were randomly divided into three groups. Eventually, the grouping scheme was kept in a sealed envelope. The patients were assigned to the three respective groups based on the grouping scheme. Both patients and researchers were unaware of fluid distribution in patients. Fluid was given to patients by a person who knew the distribution of CHO and placebo water and was not involved in the study.

**Figure 1:**
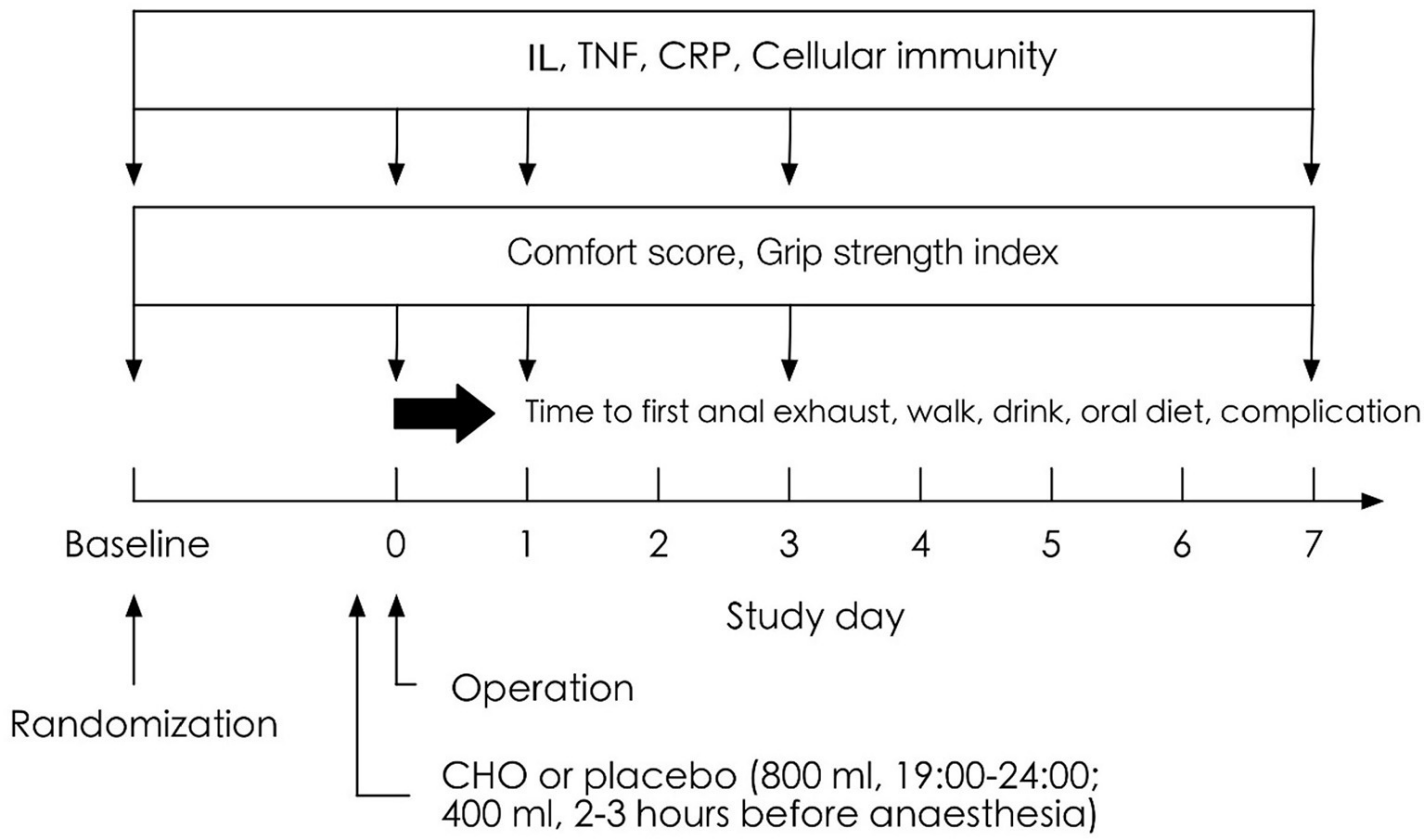
Illustration of the experimental design.

**Figure 2:**
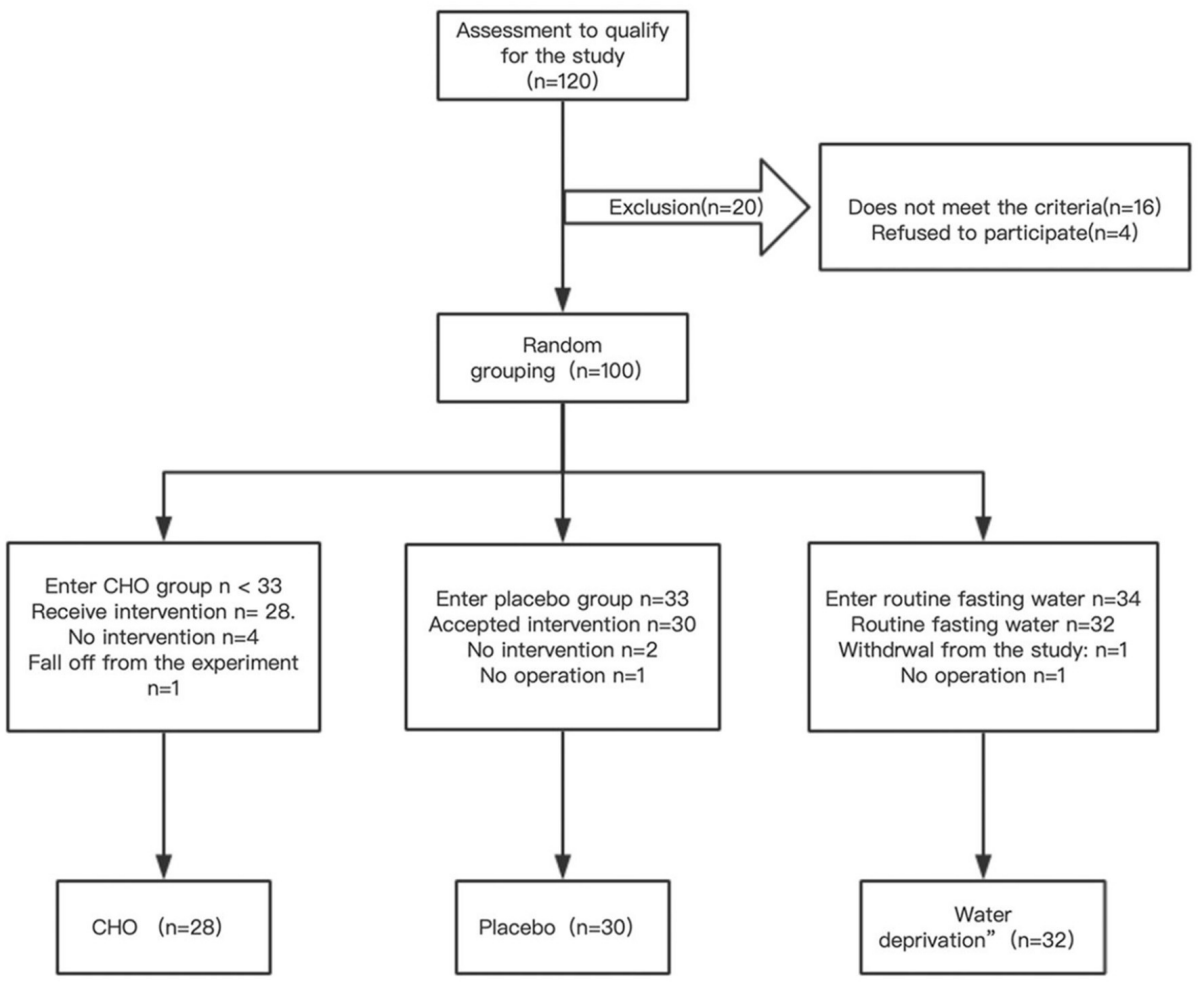
Experimental flow chart.

Patients who met the criteria were selected and randomly assigned to the CHO, water (placebo group), and routine water abstinence (fasting group) groups according to the envelope clue. CHO (Su Qian, commonly known as maltodextrin fructose drink) and placebo products were produced by Jiangsu Zhengda Fenghai Pharmaceutical Co., Ltd., and both products had the same outer packaging. After completing data entry and database locking, the company revealed the product code to the researchers. The study design is demonstrated in Figure 1, and a flow chart is demonstrated in Figure 2.

All three groups were banned from solid food at least 6 h before surgery. From 07:00 PM to 12:00 AM on the evening before surgery, patients in the CHO group were given 800 mL of a CHO drink (Su Qian contains 12.6% CHO, 50 kcal/100 mL, 290 mOsm/kg, pH 5.0, and 200 mL per bottle). On the day of surgery, patients in the CHO group consumed ~400 mL of Su Qian 2–3 h before the scheduled induction of anaesthesia, with an interval of more than 20 min. Patients in the placebo group were given the same amount of seasoning water at the same time points (sucralose, 0 kcal/100 mL; citric acid, 0 kcal/100 mL, 107 mOsm/kg, pH 5.0), which had the same taste and appearance as the CHO drink. In the fasting group, no fluid was given to patients preoperatively. To ensure smooth implementation of the experiment, the patients were scheduled for the first operation on the day of surgery. All operations were performed by the same group of experienced urological surgeons.

All patients received the same general anaesthesia regimen, with sufentanil at a dose of 0.25–0.5 μg/kg, propofol at a dose of 1.5–2 mg/kg and cisatracurium benzenesulfonate at a dose of 0.2 mg/kg. After endotracheal intubation, sevoflurane inhalation was used to maintain anaesthesia with end-expiratory sevoflurane volume fraction of 0.9–1.2 minimum alveolar concentration, remifentanil at a dose of 2–5 μg/kg/h was used to induce analgesia, and cis-atracurium besylate at a dose of 4–10 mg/h was used to maintain muscle relaxation. During surgery, fluid infusion was guided based on blood pressure, heart rate, bleeding, and urine volume. Ringer's lactate solution and hydroxyethyl starch were used as supplements, crystal fluid: Colloidal fluid = 3/1 and appropriate adjuvant vasoactive drugs were also used.

After surgery, the patients were encouraged to sit by the bedside or get out of the bed as soon as their health conditions permitted. If there was no nausea and vomiting, the patients were asked to drink water and eat as soon as possible. Infection is defined as the presence of sepsis, which can be diagnosed as follows: body temperature >38°C or <36°C, heart rate >90 beats/min, systolic blood pressure ≤ 100 mmHg, respiratory rate >22 beats min or partial pressure of carbon dioxide (PaCO_2_) <32 mmHg (<4.3 kPa), white blood cell count >12 × 10 ^∧^9/L or <4 × 10 ^∧^9/L or immature cell count >10% and changes in the consciousness level.

At ~7 AM before surgery, venous blood was collected from the patients to measure the levels of IL, tumour necrosis factor (TNF), and CRP and cellular immunity. Venous blood samples were collected at the same time point on days 1, 3, and 7 postoperatively. In addition, comfort and grip strength of the patients were measured at the same time point preoperatively and on days 1, 3, and 7 postoperatively. Comfort was measured using a 100-mm visual analogue scale (VAS) (5) based on the following parameters: anxiety, hunger, thirst, nausea, and fatigue. Grip strength was measured using a grip force device, and all measurements were performed on the same dominant hand. The first exhaust time, independent standing time after surgery, time to the intake of water and time to the intake of oral diet were recorded, and the results related to postoperative infection were assessed.

Outcome indicators included the following:

1. Main outcome indicators: levels of inflammatory markers (IL-6, IL-8, IL-10, TNF, and CRP);
2. Secondary outcomes indicators: cellular immunity level (CD3, CD4, CD8, CD4/CD8, CD19, and CD16/CD56), comfort (anxiety, hunger, thirst, nausea, and fatigue), the index of grip strength of body mass (grip strength [kg]/body weight [kg] × 100%) and clinical outcomes (first exhaust time, independent standing time after surgery, time to intake of water, time to intake of oral diet and the incidence of postoperative infection).

### Statistical Analysis

Measurement data conforming to normal distribution were represented by mean (standard deviation); non-normal distribution was represented by median (lower and upper quartiles, i.e., the interquartile range), and count data were represented by the rate of adoption (%) or composition ratio (%). Standardised differences were used to evaluate the chief demographic and clinical characteristics among different groups, and the maximum value of standardised differences between two groups compared in pairs was used as the evaluation index. The measurement data were used for repeated measures analysis of variance with adjusted covariates including Age, BMI, AT, Fluid, Blood Loss, and ASA. If the difference between the treatment groups and interaction between the repetitive (time) and treatment factors were statistically significant, multiple comparisons of the treatment factors were performed according to the measurement time points (Bonferroni method). *P* ≤ 0.05 indicated that the difference was statistically significant. IBMSPSS 20.0 was used for statistical analysis, and the statistical graphs were plotted using GraphPad Prism version 8.3.0.

## Results

Table 1 demonstrates that the three groups were well-matched in terms of age, BMI, ASA physical status classification, operative time, blood loss, and fluid rehydration. A patient in the placebo group had intraoperative bleeding of 900 mL and was infused with 1unit red blood cell suspension.

**Table 1:** Patient demographics and surgical characteristics.

**Characteristics^**a**^**	**CHO (*n =* 28)**	**Placebo (*n =* 30)**	**Fasted (*n =* 32)**	**Standardised differences^**b**^**
Age at surgery (years)	71.7 (68.5–74.5)	70.5(68.5–75.0)	70.4 (66.5–71.9)	0.421
BMI (kg/m^2^)	23.54 (22.1–26.3)	23.86 (21.4–26.28)	23.97 (23.03–25.06)	0.09
OR time (min)	152.5 (135–205)	162.5 (140–195)	154.27 (120–165)	0.387
Blood loss (mL)	200 (100–200)	200 (125–225)	200 (100–225)	0.798
Intraoperative fluid (mL) mean (min–max)	2,250 (2,000–2,250)	2,000 (1,750–2,250)	1,925 (1,750–2,000)	0.246
ASA grade (*n* [%])				0.059
I	5 (18)	6 (20)	6 (19)	
II	17 (61)	18 (60)	19 (59)	
III	6 (21)	6 (20)	7 (22)	

*CHO, Carbohydrate; BMI, body mass index; ASA, American Society of Anesthesiologists; OR, Operative*.

a *Values are expressed as median (interquartile range) for skewed distribution data or as n (%) for categorical data*.

b *Standardised difference was calculated using the R software*.

**Figure 3:**
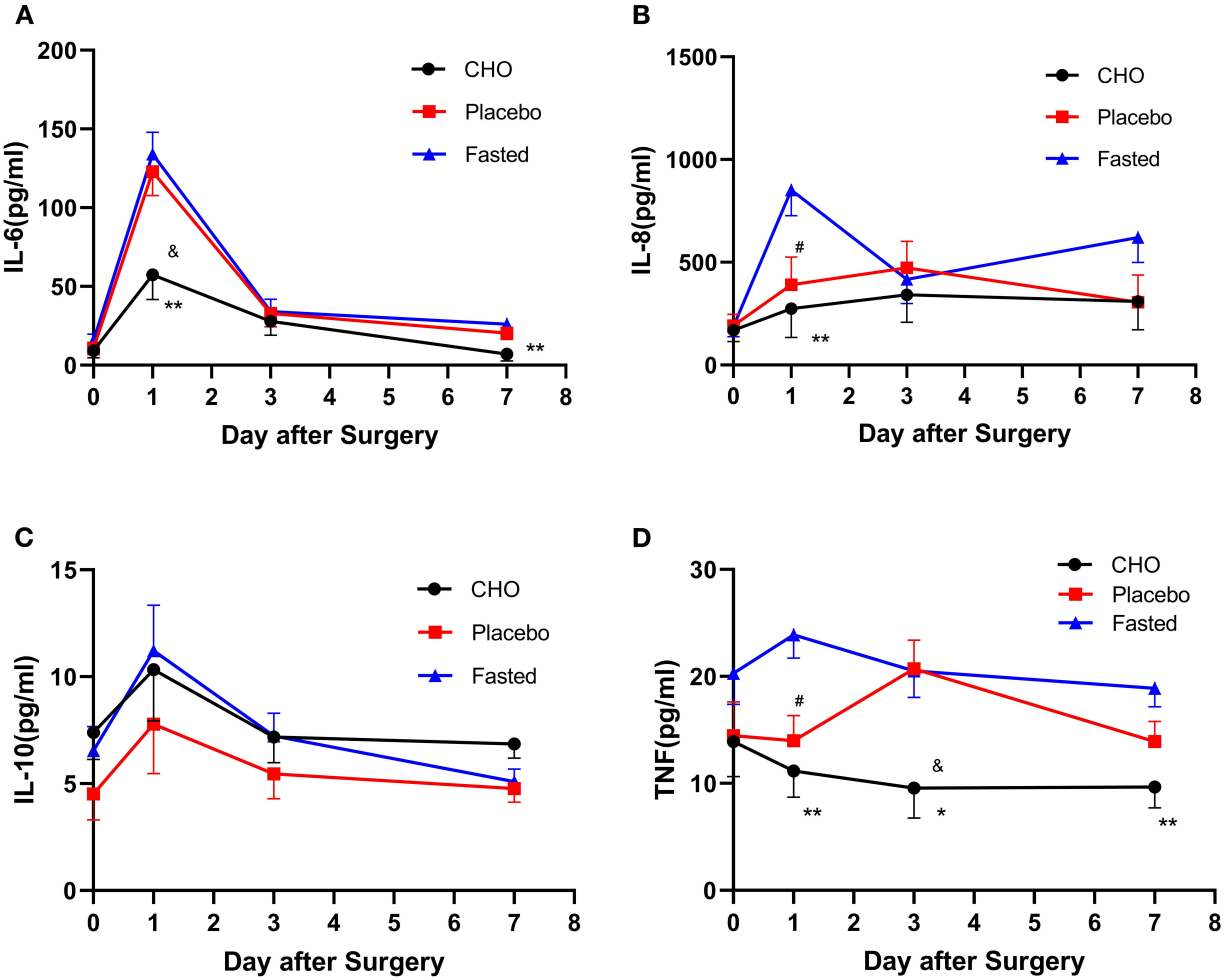
Changes in the levels of postoperative inflammatory markers including IL-6 **(A)**, IL-8 **(B)**, IL-10 **(C)**, and TNF **(D)** in different groups. CHO vs. 0.01; Placebo vs. Fasted, ^#^*P* ≤ 0.05, Placebo vs. Fasted, ^&^*P* ≤ 0.05, ^*^P ≤ 0.05, ^**^P ≤ 0.01.

Table 2, Figure 3 demonstrate the pairwise comparison of inflammatory factors at each time point in the three groups. Compared with the fasting group, the CHO group was associated with a decrease in IL-6 levels on days 1 and 7, IL-8 levels on day 1 and TNF levels on days 1, 3, and 7. Patients in the placebo group exhibited a decrease in IL-8 and TNF levels on day 1. Compared with the placebo group, the CHO group was associated with a decrease in IL-6 levels on day 1 and TNF levels on day 3. No significant difference was observed in the levels of IL-10 and CRP among the three groups. No statistical difference was observed in the cellular immune indexes among the three groups.

**Table 2:** Comparison among the levels of inflammatory factors of the three groups.

	**CHO mean (SEM)**	**Placebo mean (SEM)**	**Fasted mean (SEM)**	***P-*value**	**CHO vs. placebo mean difference (95% CI); *P-*value**	**CHO vs. fasted mean difference (95% CI); *P-*value**	**Placebo vs. fasted mean difference (95% CI); *P-*value**
**IL-6** **(pg/mL)**				0.001			
Day 0	9.3 (4.5)	11.0 (4.3)	15.8 (4.0)		−1.7 (−17.7 to 14.2); 1.000	−6.5 (−21.1 to 8.1); 0.834	−4.8 (−19.1 to 9.6); 1.000
Day 1	75.5 (15.7)	123.0 (15.1)	134 (13.9)		−65.5 (−121.654 to −9.4); **0.017**	−76.5 (−127.9 to −25.1); **0.001**	−11.0 (−61.6 to 39.5); 1.000
Day 3	27.9 (8.8)	33.0 (8.5)	34.1 (7.8)		−5.1 (−36.8 to 26.5); 1.000	−6.3 (−35.2 to 22.7); 1.000	−1.1 (−29.7 to 27.4); 1.000
Day 7	7.1 (4.3)	20.5 (4.1)	26.11 (3.8)		−13.4 (−28.8 to 1.9); 0.106	−19.1 (−33.1 to −5.0); **0.004**	−5.6 (−19.5 to 8.2); 0.973
**IL−8** **(pg/mL)**				0.011			
Day 0	170 (56)	192 (54)	188 (50)		−23 (−223 to 177); 1.000	−18.2 (−201.3 to 164.8); 1.000	4.4 (−175.6 to 184.5); 1.000
Day 1	275 (141)	390 (136)	852 (125)		−116 (−620 to 389); 1.000	−576.9 (−1038.4 to −115.4); **0.009**	−461.3 (−915.2 to −7.3); **0.045**
Day 3	341 (133)	473 (129)	417 (118)		−132 (−608 to 345); 1.000	−75.9 (−512.3 to 360.6); 1.000	55.8 (−373.5 to 485.1); 1.000
Day 7	309 (137)	305 (132)	620 (121)		4.2 (−487 to 495); 1.000	−310.8 (−760.0 to 138.4); 0.284	−315.0 (−756.8 to 126.9); 0.256
**TNF** **(pg/mL)**				0.001			
Day 0	13.9 (3.3)	14.5 (1.2)	20.3 (2.9)		−0.5 (−12.3 to 11.2); 1.000	−6.4 (−17.1 to 4.4); 0.451	−5.8 (−16.4 to 4.7); 0.541
Day 1	11.2 (2.4)	14.0 (2.4)	23.9 (2.2)		−2.8 (−11.6 to 5.9); 1.000	−12.7 (−20.7 to −4.7); **0.001**	−9.9 (−17.8 to −2.0); **0.009**
Day 3	9.6 (2.8)	20.7 (2.7)	20.5 (2.5)		−11.2 (−21.1 to −1.2); **0.023**	−11.0 (−20.1 to −1.8); **0.013**	1.2 (−8.8 to 9.2); 1.000
Day 7	9.7 (1.9)	13.9 (1.9)	18.9 (1.7)		−4.2 (−1.2 to 2.8); 0.428	−9.2 (−15.6 to −2.8); **0.002**	−5.0 (−11.3 to 1.3); 0.170

*CHO, Carbohydrate; IL−6, Interleukin 6; IL-8, Interleukin 8; SEM, Standard error of the mean; TNF, Ttumor necrosis factor; Day 0, Before the operation; Day 1, The first postoperative day; Day 3, The third postoperative day; Day 7, The seventh postoperative day. P < 0.05 is displayed in bold*.

The results of repeated measures analysis of variance revealed no interaction between IL-10 levels and measurement time points (*F* = 0.746, *P* = 0.471) among the groups. Statistical differences were observed in IL-10 levels at different time points preoperatively and postoperatively (*F* = 8.112, *P* = 0.001). No statistically significant differences were observed in IL-10 levels among the three groups (*F* = 1.148, *P* = 0.322).

Table 3 demonstrates that compared with the fasting group, the thirst (0.68 and 1.26, respectively) and hunger (0.24 and 0.47, respectively) scores of the CHO and placebo groups on the morning of surgery were significantly reduced (both *P* < 0.01). No difference was observed in the weight grip index among the three groups.

**Table 3:** Comparison among subjective comfort in the three groups.

	**CHO**	**Placebo**	**Fasted**	***P-*value (CHO vs. fasted)**	***P-*value (placebo vs. fasted)**
**Anxiety**					
Day 0	1.2 (0–8)	1.8 (0–5)	1.6 (0–4)	0.141	0.338
Day of surgery	1.0 (0–4)	1.7 (0–5)	1.2 (0–8)	0.838	0.119
Day 1	0.9 (0–7)	0.6 (0–5)	0.7 (0–4)	0.603	0.999
Day 3	0.3 (0–2)	0.2 (0–2)	0.5 (0–5)	0.339	0.365
**Thirst**					
Day 0	1.7 (0–6.5)	1.4 (0–5)	2.4 (0–5)	0.451	0.52
Day of surgery	0.7 (0–4)	1.3 (0–4)	3.0 (1–8.5)	0.002	0.001
Day 1	2.2 (0–8)	1.8 (0–8)	2.2 (0–7)	0.7	0.335
Day 3	1.2 (0–7)	0.4 (0–2)	0.8 (0–5)	0.692	0.306
**Hunger**					
Day 0	0.9 (0–3)	0.4 (0–4.5)	1.0 (0–4)	0.934	0.78
Day of surgery	0.2 (0–3)	0.5 (0–3)	1.4 (0–7)	0.008	0.01
Day 1	1.9 (0–8)	1.2 (0–5)	1.5 (0–6)	0.474	0.886
Day 3	0.8 (0–5.5)	0.4 (0–5)	0.9 (0–5)	0.889	0.439
**Nausea**					
Day 0	0 (0–0)	0.2 (0–3)	0 (0–0)	0.999	0.07
Day of surgery	1.2 (0–3)	1.5 (0–6)	2 (0–6)	0.666	0.916
Day 1	0.5 (0–5)	1.8 (0–6)	1.3 (0–3)	0.828	0.758
Day 3	0.6 (0–5)	0.1 (0–1)	0.1 (0–3)	0.138	0.585
**Fatigue**					
Day 0	1.1 (1–2)	1.0 (1–2)	1 (0–2)	0.125	0.563
Day of surgery	2.2 (0–6)	2.5 (1–6)	2.4 (0–6)	0.553	0.979
Day 1	1.6 (0–7.5)	0.9 (0–3)	1.0 (0–4)	0.128	0.786
Day 3	0.9 (0–5)	0.4 (0–3)	0.7 (0–2)	0.791	0.172
**Grip strength index (%)**					
Day 0	44.7 (29.0–67.7)	46.9 (33.4–67.7)	44.1 (30.7–62.3)	0.211	0.588
Day of surgery	38.4 (26.2–68.1)	41.5 (32.8–57.5)	41.3 (25.9–57.9)	0.57	0.475
Day 1	39.2 (27.6–66.0)	42.1 (24.8–63.2)	43.0 (30.9–61.2)	0.968	0.567
Day 3	42.4 (29.7–66.1)	44.1 (25.1–63.1)	44.1 (28.8–64.9)	0.496	0.691

*CHO, Carbohydrate; Day 0, Before the operation; Day 1, The first postoperative day; Day 3, The third postoperative day; Day 7, The seventh postoperative day*.

Table 4 demonstrates no differences in independent standing time, the first exhaust time, the first water intake time, the first mealtime and the incidence of postoperative infection among the three groups.

**Table 4:** Comparison among the postoperative rehabilitation indices of the three groups.

	**CHO mean (min–max)**	**Placebo mean (min–max)**	**Fasted mean (min–max)**	**CHO vs. placebo mean difference (95% CI); *P–*value**	**CHO vs. fasted mean difference (95% CI); *P-*value**	**Placebo vs. fasted mean difference (95% CI); *P-*value**
Time to first anal exhaust (h)	24.7 (5.5–101)	26.5 (7.5–64)	25.0 (1.5–93)	−1.8 (−11.2 to 7.7); 1.000	−0.32 (−9.30 to 8.65); 1.000	1.44 (−7.91 to 10.79); 1.000
Time to first walk (h)	28.6 (10–100)	28.7 (12–70)	39.2 (15–93)	−1.4 (−10.2 to 9.9); 1.000	−10.64 (−20.03 to −1.25); 0.081	−10.50 (−20.29 to −0.712); 0.108
Time to first drink (h)	24.7 (5.5–101)	26.5 (7.5–64)	25.0 (1.5–93)	−3.9 (−4.6 to 3.8); 1.000	−1.97 (−5.87 to 1.92); 0.948	−1.58 (−5.69 to 2.53); 1.000
Time to start oral diet (h)	27.9 (12–73)	34.8 (8–72)	34.6 (2.5–80)	−6.9 (−16.5 to 2.7); 0.474	−6.65 (−15.72 to 2.40); 0.441	0.22 (−9.68 to 9.24); 1.000
**Infection (** ***n*** **)**						
Day 1	4	4	3	1.000	1.000	1.000
Day 3	2	2	3	1.000	1.000	1.000
Day 7	0	0	0	1.000	1.000	1.000

*CHO, Carbohydrate; Day 0, Before the operation; Day 1, The first postoperative day; Day 3, The third postoperative day; Day 7, The seventh postoperative day*.

*CHO vs. fasted*.

## Discussion

Indicators for the clinical evaluation of immune function include inflammatory markers (IL-2, IL-6, IL-8, IL-10, TNF, and CRP) (11) and cellular immunity (T cells, T helper cells, natural killer [NK] cells, and human leukocyte antigens DR [HLA-DR]). Of these inflammatory factors, IL-6, IL-8, TNF, and CRP are all pro-inflammatory factors, and some studies have reported that IL-10 is an anti-inflammatory factor. A decrease in the levels of inflammatory markers and an increase in cellular immunity indicate that an individual's immune function is better (12, 13). To reduce the occurrence of postoperative complications, studies have suggested that accelerated rehabilitation surgery, especially minimally invasive surgery (14), and unconventional fasting before surgery can improve postoperative immune function, reduce inflammation levels, and increase cell-mediated specificity. In 2006, the Gerdien et al. (15) investigated the effect of preoperative liquid CHO intake on postoperative immune function. Compared with the routine preoperative water deprivation group, the HLA-DR levels of the oral CHO group did not decrease and body fluid balance was not disturbed, indicating that preoperative oral administration of CHO can avoid subsequent immune reactions and reduce the incidence of complications such as infection. However, another study by Mathur et al. demonstrated that CRP and IL-6 levels exert no effect on systemic inflammation in patients undergoing major abdominal surgery (16). Therefore, the authors believed that there is no evidence that CHO load is essential to reduce surgical pressure. Tran et al. (17) found that the levels of IL-6 and CRP were not affected by the use of CHO before coronary artery bypass grafting and spinal surgery.

This study revealed that the levels of inflammatory markers in the placebo and CHO groups were lower than those of the fasting group; the levels were especially lower in the CHO group. Compared with the fasting group, the CHO group exhibited a decrease in TNF levels on days 1, 3, and 7 postoperatively, IL-6 levels on days 1 and 7 postoperatively and IL-8 levels on day 1 postoperatively. Compared with the fasting group, the placebo group exhibited a decrease in IL-8 and TNF levels on the first postoperative day. The levels of three major inflammatory factors (IL-6, IL-8, and TNF) were significantly reduced on the first postoperative day, indicating that CHO was closely associated with decreased levels of inflammatory markers. The levels of two inflammatory factors (IL-8 and TNF) in the placebo group were also significantly reduced on the first postoperative day, indicating that drinking water was also associated with the reduction of inflammatory factors. Compared with the placebo group, the CHO group only exhibited a decrease in IL-6 levels on the first postoperative day and TNF levels on the third postoperative day, indicating that CHO did not offer many advantages to reduce the levels of inflammatory factors. Therefore, preoperative consumption of a certain amount of liquid, whether CHO, sweet water, or other clear liquids, exerts similar effects on postoperative inflammation indicators. Su Qian is an energy-rich CHO beverage, whereas water is a transparent liquid without energy-rich nutrients; the difference between Sugan and water is that their sugar and energy contents are 1 and 0, respectively. Sugar and energy may not play an important role in regulating the level of inflammatory markers, and a certain amount of fluid intake preoperatively may exert significant effects on postoperative results. Compared with water deprivation, preoperative intake of a certain amount of fluid can significantly reduce the levels of inflammatory markers in the body.

Anti-inflammatory cytokines are immunoregulatory molecules that control the pro-inflammatory cytokine response. They interact with specific cytokine inhibitors and soluble cytokine receptors to regulate the human immune response. Their physiological role in inflammation and pathological role in systemic inflammatory states are increasingly being recognised. Major anti-inflammatory cytokines include IL-1 receptor antagonist, IL-4, IL-10, IL-11, and IL-13. Of all anti-inflammatory cytokines, IL-10 exhibits potent anti-inflammatory properties, repressing the expression of inflammatory cytokines such as TNF-α, IL-6 and IL-1 by activated macrophages. In addition, IL-10 can upregulate endogenous anti-cytokines and downregulate pro-inflammatory cytokine receptors. Therefore, it can counter-regulate the production and function of pro-inflammatory cytokines at multiple levels (18).

In this study, no statistical difference was observed in IL-10 levels among the three groups, indicating that the effect of preoperative liquid intake was weaker on anti-inflammatory factors such as IL-10 than that on pro-inflammatory factors such as IL-6, IL-8, and TNF.

Although significant differences were observed in the levels of IL-6, IL-8, and TNF among the three groups, no difference was observed in the levels of cellular immunity indicators and the incidence of postoperative infection among the three groups; therefore, the clinical significance of CHO administration could not be determined. Because several factors affect the incidence of postoperative infection, preoperative fluid intake may not be a key factor in reducing the incidence of postoperative infections.

Several studies (19) have demonstrated that preoperative administration of CHO can significantly reduce preoperative hunger and anxiety and does not affect gastric volume. The present study found that preoperative administration of fluid, either CHO or clear liquid, can significantly improve thirst and hunger scores in the early morning (usually 90–120 min after the intake of liquid in the morning) as compared with water deprivation. A similar effect was observed on the comfort parameters on the day of surgery. Furthermore, drinking a liquid beverage, not necessarily CHO, may significantly provide improved comfort to patients.

Clinical outcomes among the three groups were not significantly different. Preoperative liquid intake did not play an important role because several factors affect the clinical outcome of patients. For example, the time to get out of bed is affected by factors such as medical staff education, medical cognition update, fear of getting out of bed, postoperative pain, and weakness. Some studies (20) have reported that preoperative CHO load is only related to a small reduction in the length of hospital stay and exerts no effect on the incidence of complications. In China, the length of hospital stay is affected by various factors; therefore, the clinical results of this study do not include postoperative hospital stay. Compared with a study conducted by Veenhof et al. (14), this study included a group of placebo controls. Compared with a study conducted by Mathur et al. (16), this study included a set of blank controls. This study demonstrated that CHO and placebo almost offer the same advantages in reducing the levels of inflammatory markers; however, no significant difference was observed in the incidence of postoperative infection among the three groups.

This quality study has some limitations. The sample size was small, and the level of inflammatory markers was not necessarily associated with the incidence of infection. Therefore, it is necessary to further investigate the influence of CHO or clear liquid on the inflammatory markers and clinical outcomes of elderly patients undergoing major surgery.

## Conclusions

Preoperative CHO and drinking water are associated with decreased levels of IL-6, IL-8, and TNF. CHO and water can also reduce thirst and hunger scores. Therefore, we recommend that patients without contraindications should be given 200–400 ml of fluid 2–3 h before surgery, preferably CHO.

## Data Availability Statement

The original contributions presented in the study are included in the article/supplementary materials, further inquiries can be directed to the corresponding author/s.

## Ethics Statement

The studies involving human participants were reviewed and approved by the ethical standards of Shanghai Tenth People's Hospital and with the 1964 Helsinki declaration and its later amendments or comparable ethical standards. The patients/participants provided their written informed consent to participate in this study. Written informed consent was obtained from the individual(s) for the publication of any potentially identifiable images or data included in this article.

## Author Contributions

ZH planned and implemented the experiment and wrote the article. JL retouched, revised, and contributed to the article. FW supervised the experiment and supported it with financial support. All authors contributed to the article and approved the submitted version.

## Funding

This study was funded by the Shanghai Science Committee Foundation (Grant Number, 19411967700) and the Natural Science Foundation of China (Grant Number, 81472389 and 81672549).

## Conflict of Interest

The authors declare that the research was conducted in the absence of any commercial or financial relationships that could be construed as a potential conflict of interest.

## Publisher's Note

All claims expressed in this article are solely those of the authors and do not necessarily represent those of their affiliated organizations, or those of the publisher, the editors and the reviewers. Any product that may be evaluated in this article, or claim that may be made by its manufacturer, is not guaranteed or endorsed by the publisher.

## References

[1] Bilku DK Dennison AR Hall TC Metcalfe MS Garcea G. Role of preoperative carbohydrate loading: a systematic review. Ann R Coll Surg Engl. (2014) 96:15–22. 10.1308/003588414X13824511650614 24417824 PMC5137663

[2] Weimann A Braga M Harsanyi L Laviano A Ljungqvist O Soeters P. ESPEN guidelines on enteral nutrition: surgery including organ transplantation. Lin Nutr. (2006) 25:224–44. 10.1016/j.clnu.2006.01.015 16698152

[3] Feldheiser A Aziz O Baldini G Cox BPBW Fearon KCH Feldman LS. Enhanced Recovery After Surgery (ERAS) for gastrointestinal surgery, part 2: consensus statement for anaesthesia practice. Acta Anaesthesiol Scand. (2016) 60:289–334. 10.1111/aas.12651 26514824 PMC5061107

[4] Kehlet H Wilmore DW. Multimodal strategies to improve surgical outcome. Am J Surg. (2002) 183:630–41. 10.1016/S0002-9610(02)00866-8 12095591

[5] Hausel J Nygren J Lagerkranser M Hellström PM Hammarqvist F Almström C. A carbohydrate-rich drink reduces preoperative discomfort in elective surgery patients. Anesth Analg. (2001) 93:1344–50. 10.1097/00000539-200111000-00063 11682427

[6] Noblett SE Watson DS Huong H Davison B Hainsworth PJ Horgan AF. Pre-operative oral carbohydrate loading in colorectal surgery: a randomized controlled trial. Colorectal Dis. (2006) 8:563–9. 10.1111/j.1463-1318.2006.00965.x 16919107

[7] Dilmen OK Yentur E Tunali Y Balci H Bahar M. Does preoperative oral carbohydrate treatment reduce the postoperative surgical stress response in lumbar disc surgery? Clin Neurol Neurosurg. (2017) 153:82–6. 10.1016/j.clineuro.2016.12.016 28073036

[8] Zhang Y Min J. Preoperative carbohydrate loading in gynecological patients undergoing combined spinal and epidural anesthesia. J Invest Surg. (2019) 33:587–95. 10.1080/08941939.2018.1546352 30644785

[9] Porcaro AB de Luyk N Corsi P Sebben M Tafuri A Inverardi D. Robotic assisted radical prostatectomy accelerates postoperative stress recovery: final results of a contemporary prospective study assessing pathophysiology of cortisol peri-operative kinetics in prostate cancer surgery. Asian J Urol. (2016) 3:88–95. 10.1016/j.ajur.2016.03.002 29264170 PMC5730811

[10] Pedziwiatr M Pisarska M Matłok M Major P Kisielewski M Wierdak M. Randomized clinical trial to compare the effects of preoperative oral carbohydrate loading versus placebo on insulin resistance and cortisol level after laparoscopic cholecystectomy. Pol Przegl Chir. (2015) 87:402–8. 10.1515/pjs-2015-0079 26495916

[11] Akdis M Aab A Altunbulakli C Azkur K Costa RA Crameri R. Interleukins (from IL-1 to IL-38), interferons, transforming growth factor β, TNF-α: receptors. functions, and roles in diseases. J Allergy Clin Immunol. (2016) 138:984–1010. 10.1016/j.jaci.2016.06.033 27577879

[12] Wu FPK Sietses C von Blomberg BME van Leeuwen PAM Meijer S Cuesta MA. Systemic and peritoneal inflammatory response after laparoscopic or conventional colon resection in cancer patients: a prospective, randomized trial. Dis Colon Rectum. (2003) 46:147–55. 10.1007/s10350-004-6516-2 12576886

[13] Schwenk W Jacobi C Mansmann U Böhm B Müller JM. Inflammatory response after laparoscopic and conventional colorectal resections-results of a prospective randomized trial. Langenbecks Arch Surg. (2000) 385:2–9. 10.1007/s004230050002 10664112

[14] Veenhof AAFA Vlug MS van der Pas MHGM Sietses C van der Peet DL de Lange-de Klerk ESM. Surgical stress response and postoperative immune function after laparoscopy or open surgery with fast track or standard perioperative care: a randomized trial. Ann Surg. (2012) 255:216–21. 10.1097/SLA.0b013e31824336e2 22241289

[15] Melis GC van Leeuwen PA von Blomberg-van der Flier BM Goedhart-Hiddinga AC Uitdehaag BM Strack van Schijndel RJ. A carbohydrate-rich beverage prior to surgery prevents surgery-induced immunodepression:a randomized, controlled,clinical trial. JPEN J Parenter Enteral Nutr. (2006) 30:21–6. 10.1177/014860710603000121 16387895

[16] Mathur S Plank LD McCall JL Shapkov P McIlroy K Gillanders LK. Randomized controlled trial of preoperative oral carbohydrate treatment in major abdominal surgery. Br J Surg. (2010) 97:485–94. 10.1002/bjs.7026 20205227

[17] Tran S Wolever TM Errett LE Ahn H Mazer CD Keith M. Preoperative carbohydrate loading in patients undergoing coronary artery bypass or spinal surgery. Anesth Analg. (2013) 117:305–13. 10.1213/ANE.0b013e318295e8d1 23757474

[18] Zhang JM An J. Cytokines, inflammation, and pain. Int Anesthesiol Clin. Spring. (2007) 45:27–37. 10.1097/AIA.0b013e318034194e 17426506 PMC2785020

[19] Çakar E Yilmaz E Çakar E Baydur H. The effect of preoperative oral carbohydrate solution intake on patient comfort: a randomized controlled study. J Perianesth Nurs. (2017) 32:589–99. 10.1016/j.jopan.2016.03.008 29157765

[20] Watt DG McSorley ST Horgan PG McMillan DC. Enhanced recovery after surgery: which components, if any, impact on the systemic inflammatory response following colorectal surgery?: A systematic review. Medicine. (2015) 94:e1286. 10.1097/MD.0000000000001286 26356689 PMC4616657

